# Endourology Methods in Pediatric Population for Kidney Stones Located in Lower Calyx: FlexURS vs. Micro PCNL (MicroPERC®)

**DOI:** 10.3389/fped.2021.640995

**Published:** 2021-05-21

**Authors:** Adam Halinski, Henri Steyaert, Magdalena Wojciech, Bartłomiej Sobolewski, Andrzej Haliński

**Affiliations:** ^1^Department of Pediatric Urology, Private Medical Center “Klinika Wisniowa”, Zielona Góra, Poland; ^2^Department of Clinical Genetics and Pathomorphology, Collegium Medicum, University of Zielona Góra, Zielona Góra, Poland; ^3^Department of Pediatric Surgery, Queen Fabiola Children's University Hospital, Université Libre de Bruxelles (ULB), Brussels, Belgium; ^4^Department of Mathematical Statistics and Econometrics, Faculty of Mathematics, Computer Science and Econometrics, University of Zielona Góra, Zielona Góra, Poland

**Keywords:** endourology, children, kidney stone disease, flexible ureterenoscopy, micropercutaneous, lower calyceal calculus

## Abstract

Kidney stone disease in children is always a therapeutic challenge. It is a multifactorial condition and it should be approached, diagnosed and treated as such. One of the biggest challenges is kidney stones located in the lower renal calyx. There are currently three main surgical techniques to treat this condition: ESWL—Extracorporeal Shock Wave Lithotripsy, RIRS—Retrograde IntraRenal Surgery, and PCNL—PerCutaneous Nephro-Lithotripsy. In pediatric population, the most frequently used method is ESWL, and in the event of failure, endoscopic procedures are the second-best choice. In this article, a sample of 53 children admitted to a tertiary medical center was examined. Thirty-eight of those children underwent flexible URS, while the remaining 15—micro PCNL. The average size of the deposit in the former group was 12.2 mm, against 13.5 mm in the latter. The full Stone Free Rate (SFR) was achieved in RIRS at 84.21 and 86.7% in percutaneous nephrolithotripsy. Flexible ureterorenoscopy and MicroPERC are two comparably effective methods for treating lower calyx stones of any size. However, according to our data, flexible ureterorenoscopy carries a lower risk of complications and inpatient care (with the mean of 3 days). The learning curve for these procedures in pediatric urology is long and relies on a limited number of patients. The number of pediatric patients qualifying for these procedures is restricted also due to the high efficacy of extracorporeal shock wave lithotripsy in pediatric population. Radiation exposure is an important factor in every endoscopy procedure and should never exceed the limits set in the ALARA protocol. ESWL remains to this day the treatment of choice for stone disease in children and can be performed under ultrasound control. For many parents, it is a first-choice treatment preference for their child due to its greater apparent safety, although data on this remains insufficient. Prospective, randomized, multicenter trials are definitely needed.

## Introduction

Deposits located in the lower renal calyx in both adults and children are always a challenge for urologists. While kidney stone disease is a relatively common condition in adults that affects from 3 to 5% of the population (1), it is noteworthy—from the epidemiological point of view—that children comprise only 2–3% of all urolithiasis patients (2, 3). On the other hand, the number of patients suffering from kidney stone disease increases every year. Treatment should therefore be individualized, while the patient and their parents should be informed about all available treatment options, which are: extracorporeal shock wave lithotripsy (ESWL), flexible ureterorenoscopy (Flex URS, RIRS), percutaneous nephrolithotripsy (PCNL). There are numerous scientific publications that describe contraindications to the ESWL procedure, namely: a small lower pole infundibulo-pelvic angle (LIPA), a tight infundibular width and a long infundibular length (4, 5). Other authors, meanwhile, argue that only the size of the deposit (<1 cm = 74%, 1–2 cm = 56%, and more than 2 cm = 33%) has an impact on the stone free rate (6). However, as shown by our statistical and clinical observations, parents opt for the ESWL treatment because they consider it safer and the least invasive of all (7). In the case of lack of consent, failure or contraindications for the ESWL procedure, two endoscopic alternatives can be proposed: transurethral RIRS or percutaneous PCNL. Due to the progressing miniaturization of the equipment, the diameter of the instruments in both cases enables intervention in increasingly younger pediatric patients (8). In our practice, the youngest patient who successfully underwent the RIRS procedure was a 8 month-old girl with bilateral kidney stone disease. For the PCNL procedure, we use the MicroPERC® system in which the telescope, working channel and irrigation are combined into a needle with the size of 4.8 Fr. The procedure requires only a single puncture, thereby avoiding the need for tract dilatation (9).

## Materials and Methods

Data was collected prospectively and assessed retrospectively. Study started on June 2015 and ended in August 2016. In this study we include 53 pediatric patients with stone located in lower calyx. Of these, 38 (22 girls and 16 boys) underwent the RIRS procedure and 15 (10 girls and 5 boys)—the MicroPERC procedure. In the preparation protocol we perform ultrasound examination, or in selected cases CT scans without contrast (low dose NCCT). If the patient was prepared for kidney puncture the prone position during CT was used. Parents and patients were fully informed about the course of the procedure, potential complications, the post-operative DJ catheter stent, and the likelihood of a second-look endoscopy/open procedure. Urine culture was mandatory before the surgery [when aseptive inoculation—antibiotic prevention: cefuroxime; patients with asymptomatic bacteriuria (ASB)—treated according to the antibiogram at least 3 days before the surgery; feverish patients—procedure postponed]. Blood tests carried out were: morphology, coagulation tests, serum creatine, and electrolytes. We qualified 33 patients after unsuccessful ESWL surgery and 20 patients, with stones in the lower calyx, above 1 cm and below 2 cm. Four children qualified for MicroPERC underwent unsuccessful RIRS intervention. Reason for that was small renal pelvis, which restricted the deflection of the flexible endoscope.

Patients were split into two groups (Group 1 = Flex URS/RIRS, Group 2 = MicroPERC). In the first group, the age of patients ranged from 18 months to 18 years (SD ± 3.78, mean = 9.6 years). Stones varied in size from 1 to 1.5 cm, with the mean of 1.35 cm. In the second group, the age of patients ranged from 3 years to 16 years (SD ± 3.36, mean = 8.2 years). Stones in this group measured from 1 to 1.6 cm, with the mean of 1.22 cm. The irrigation system was only gravity (situated 30 cm above the patient) in the first and second cohort. Saline irrigation was carefully monitored during the procedure. In both groups, the laser device Quanta System Litho® 2000 and a 272 μm laser fiber were used. Fluoroscopy C-arm was placed over the patient from the begging of the procedure, ready to be used at all times.

**Figure 1:**
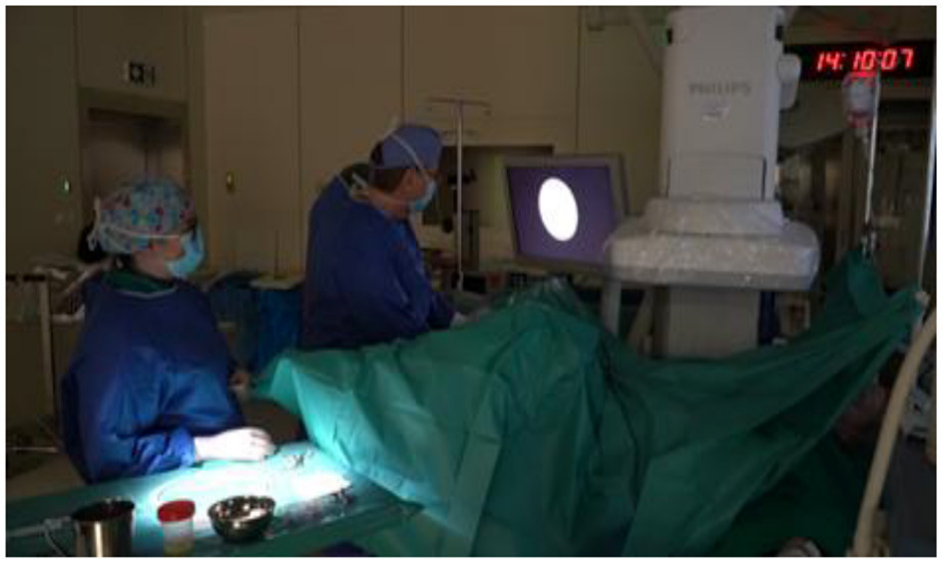
Flexible ureterorenoscopy in hybrid operating theater (OT).

**Figure 2:**
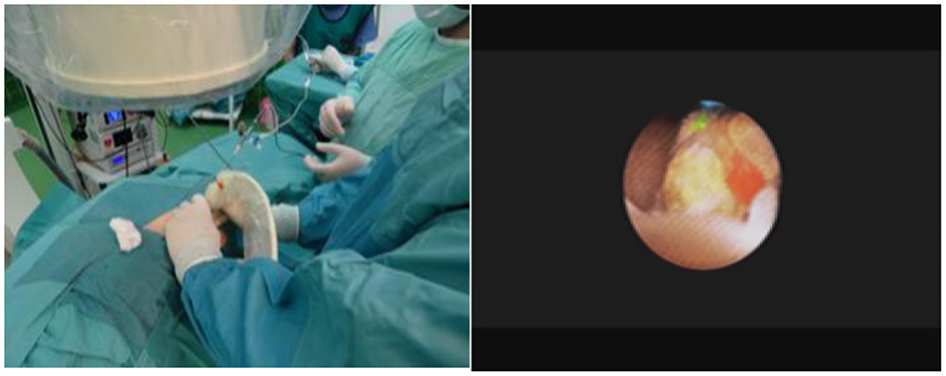
Kidney puncture under ultrasound control. Stone in the “all-seeing needle” view.

For RIRS procedure (Figure 1), 7.5 Fr flexible ureterorenoscope from STORZ® company and 9.5 Fr ureteral access sheath were used. All patients were pre-stented 2 weeks prior to the procedure, allowing for 100% ureteral access without the need for ureteral meatus dilatation. After RIRS, a DJ stent was placed for 2 weeks. Foley catheter was removed the following day, in the morning hours. No renal colic was observed. We have been performing flexible ureterorenoscopy in pediatric population since 2013. In second group, we used the MicroPERC® equipment with the size of 4.8 Fr (Figure 2). Patients were placed in the prone position. Puncture was performed by the urologist using an “all-seeing needle” only under ultrasound control. Following the procedure, a single-J ureteral catheter and Foley was placed for 24 h. No nephrostomy tube was left inside, thus making it a tubeless surgery. We have been performing micro PCNL—MicroPERC® since 2015.

Outcomes of the surgeries were divided into 3 groups depending on their efficacy. Group A: complete removal of the stone and no complications. Group B: residual fragments up to 2 mm and no complications. In the last Group C we included patients with resistant stones larger than 2 mm or/with concomitant complications.

A statistical analysis of correlations between the groups, the efficacy of surgical approaches and the sex of the patient were tested using either Pearson's chi-squared test of independence or Fisher's exact test. Fisher's exact test of independence was used when the assumptions of the chi-square test did not hold. In order to compare the mean numerical variable for two groups of independent samples, Student's *t*-test was used. Goodness of fit with the normal distribution was verified using Shapiro-Wilk's test. When the data was distributed normally, the equality of variance was tested with Bartlett's test. The Wilcoxon rank-sum test with continuity correction was used when the assumptions of normal distribution did not hold. In order to control the number of false positive results in a series of tests for comparing two means, the false discovery rate (FDR) method was used. By using Benjamini-Hochberg's FDR, we were able to adjust the *p*-value in multiple testing.

For all the statistical tests, the level of statistical significance was set at 0.05. The analyses were conducted using the R 4.0.3 software (10).

## Results

In the RIRS group, the episode of inpatient care ranged from 2 to 5 days (with the mean of 3 days). The mean fluoroscopy time during the procedure was 20 s.

In the MicroPERC group, the episode of inpatient care ranged from 4 to 7 days (with the mean of 4.5 days). The mean fluoroscopy time during the PCNL was 12 s.

**Figure 3:**
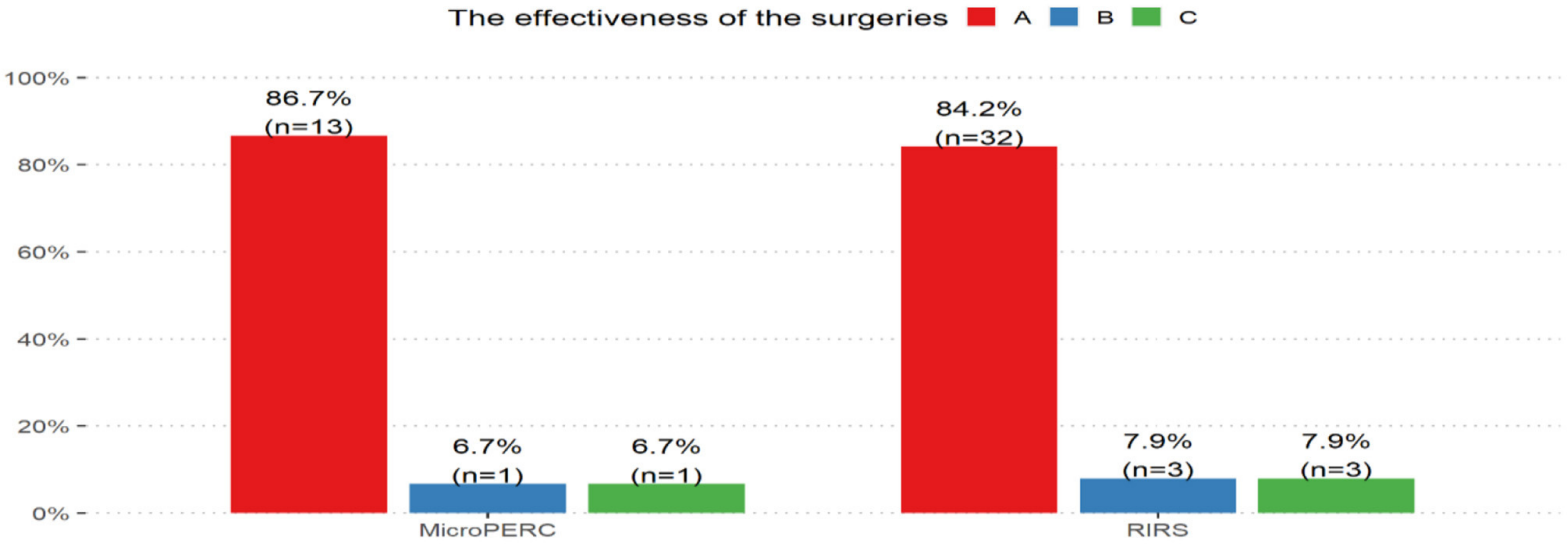
The effectiveness of the surgeries.

The stone free rate after a single procedure was 84.21% for flexible ureterorenoscopy and 86.7% for the MicroPERC method. According to Fisher's exact test, no significant difference between the efficacy in both groups was found (*p* = 1.000). The distribution of the efficacy of surgical approaches for the two methods of procedures is shown in Figure 3.

**Figure 4:**
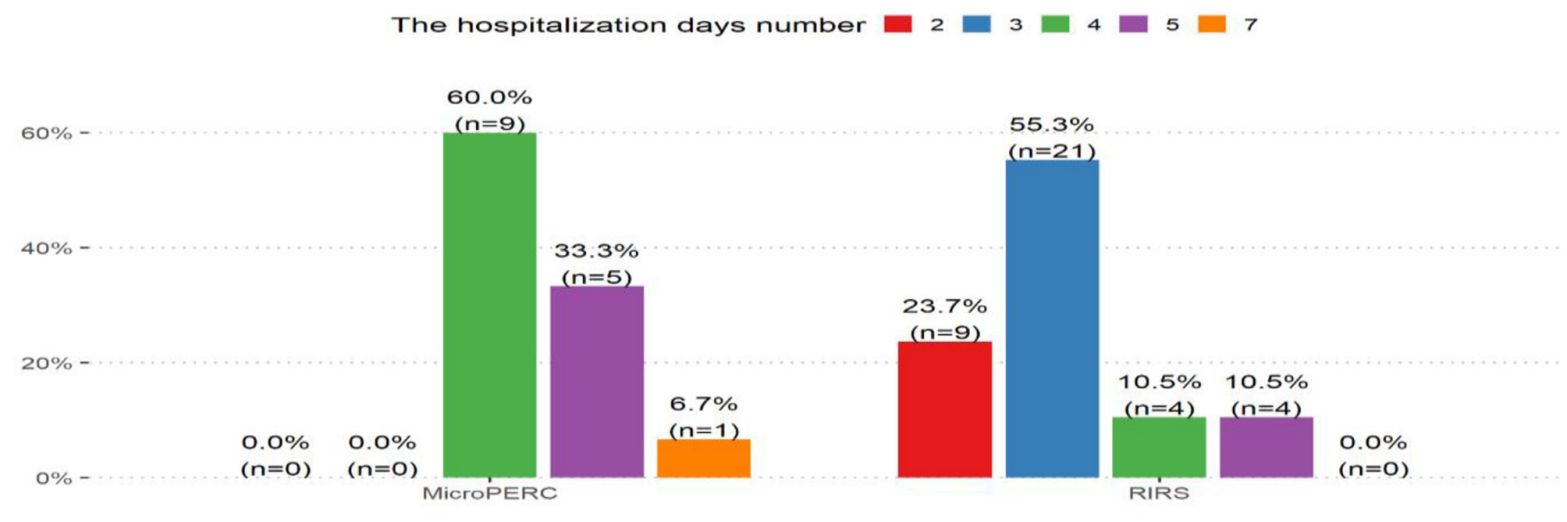
The hospitalization days number.

Based on Wilcoxon's test, we conclude that the time of hospitalization in the RIRS group was statistically shorter than in the MicroPERC group (*p* < 0.001). The distribution of the hospitalization days number for these two methods is shown in Figure 4. On the other hand, the time of fluoroscopy was longer for the flexible ureterorenoscopy procedure (*p* < 0.001). An important observation from our data, based on fluoroscopy time during RIRS, is that along with the increase of experience, the time of the procedure decreases on a case-to-case basis. It was also found that stones were larger in the MicroPERC group (*p* = 0.016).

On the basis of Student's *t*-test, no statistically significant difference was found in the mean time of lithotripsy (*p* = 0.565) and patient age (*p* = 0.2851). The chi-square independence test did not show any statistically significant correlations between the sex of the patient and the treatment procedure used (*p* = 0.7822). No major complications were observed in any of the groups. Minor complications (hematuria, renal colic), according to the Clavien three-point grading system, were: 6% for the PCNL and 2.6% for the RIRS group.

## Discussion

The number of pediatric patients with kidney stone disease increases every year. Along with it, the location of stones in the urinary system also changes. Deposits in pediatric population used to be located mainly in the urinary bladder, whereas now there is an increasingly larger number of them being diagnosed in the upper urinary tract (11). The age at which children experience kidney stones varies, but ranges mostly from 5 to 15 years, according to most data (12). Assessment (diagnostic, metabolic, genetic) of nephrolithiasis in children differs from that performed for adults. The non-typical symptoms in pediatric population require a high level of clinical knowledge. Imaging must be performed with care, to identify stones and avoid unnecessary radiation exposure (13). The ALARA protocol limits must always be observed. According to the high recurrence rate in pediatric population, the probability of further, repeated diagnostic images has increased.

We would like to compare our results with those from the literature. Due to limited input, there is still not enough data indicating the successful application of one given method in children patients. However, based on the accessible data, it can be observed that in stones with the size from 1 to 2 cm, the success rate of RIRS and PCNL is comparable (14, 15). Meanwhile, in stones larger than 2 cm, PCNL provides better outcomes in SFR with 71 to even 95% (16).

The size of the laser fiber, which enables the best deflection with the smallest damage of the flexible scope and which can also be placed into the 4.8 Fr “all seeing needle” device, is 272 μm. In this sense, these two techniques are similar. In our view, however, there is indeed potential for faster lithotripsy in the newest types of laser devices. Efforts are underway to reduce lithotripsy time as well as to limit retropulsion and fiber tip degradation (17).

In our data, longer episodes of inpatient care are associated with the PNL technique. Interestingly, the same findings can be seen in Resorlu et al. (18) and Baş et al. (15).

The complication rate in MiniPERC in our paper was 6%, which is lower than in the literature (15). Comparing size of the equipment and complications, in a multicentre retrospective analysis, this rate can reach values as high as 27.7% with Mini PCNL (19).

We chose to leave DJ stents in all patients who underwent RIRS. No renal colic complications were observed, whereas the maximum time of stent placement was 2 weeks. Time is a crucial factor here due to the possible encrustation of the catheters even after a few days (20). DJ stent was removed as a procedure of a 1 day surgery.

The flexible ureterorenoscopy and MicroPERC methods are both a feasible and effective treatment for stones in the lower calyx in pediatric population (15, 21). Sometimes, due to the small size of the renal pelvis, issues with endoscope deflection may not allow the surgeon to intervene in the lower calyx (22). We have noticed, however, that parents are unlikely to opt for percutaneous surgeries if they have access to transurethral procedures, which is in line with the findings shared in Smaldone et al. (23). As it turns out, in this aspect not much has changed. Parents and patients should be informed about the available treatment options and of their efficacy, safety and complications (15).

Last but not least, it is important to establish the cause of nephrolithiasis in children. Due to the risk factors and significant rate of recurrence in pediatric population, all children must receive a complete assessment including the metabolic (13), genetics, infections and anatomical workup.

## Conclusions

Flexible ureterorenoscopy and MicroPERC are comparably effective methods of treating lower calyx stones of any size. However, the former carries a lower risk of complications and inpatient care as per our data. The learning curve for performing these procedures in pediatric urology is long and radiation exposure allowed by the ALARA protocol must never be exceeded. The number of children qualifying for these procedures is limited also due to the high efficacy of extracorporeal shock wave lithotripsy in pediatric population. ESWL remains to this day the treatment of choice for stone disease in children (12). For many parents, it is a first-choice treatment preference for their child due to its greater apparent safety. However, ECIRS is an option for patients with a small renal pelvis, although data on this for pediatric population is still insufficient (24). Prospective, randomized, multicenter trials are definitely needed.

## Data Availability Statement

The original contributions presented in the study are included in the article/supplementary material, further inquiries can be directed to the corresponding author/s.

## Author Contributions

All authors listed have made a substantial, direct and intellectual contribution to the work, and approved it for publication.

## Conflict of Interest

The authors declare that the research was conducted in the absence of any commercial or financial relationships that could be construed as a potential conflict of interest.
